# Supplementary material for: Sloppiness: Fundamental study, new formalism and its application in model assessment
Source: PLoS One. 2023 Mar 8;18(3):e0282609. doi: 10.1371/journal.pone.0282609 (PMC9994762; doi:10.1371/journal.pone.0282609)
Supplement: S1 File — This file contains the analysis of two more examples (a) High dimensional biochemical pathway to demonstrate the efficacy of the method of handle large models (b) A Cholesterol absorption model to demonstrate the ability to detect local structural unidentifiability of all the parameters. (PDF) [file pone.0282609.s001.pdf]

Sloppiness: fundamental study, new formalism and its application in model assessment

Additional Examples

September 2022

1 A pharmacokinetc model cholesterol distribution and turnover

In this example we consider a two compartment pharmacoknetic model given in [1]. This model has been widely used to represent the dynamics of distribution and turnover of labeled cholesterol in humans with impulse injections and then concentration is measure in compartment one [2]. The model has two states, four parameters (Fixing the volume of the compartment (V) to unity) and one output. The model diagram is given in Fig. 1 and equations are given in (1).

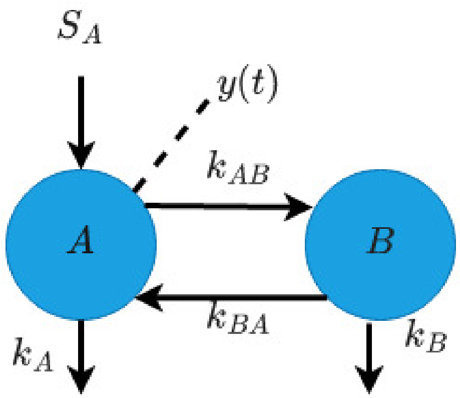

Figure 1: Two compartmental model for cholesterol distribution and turnover

$$\mathcal{M}: \begin{cases} \dot{A}(t) = -(k_A + k_{BA})A(t) + k_{AB}B(t) + S_A(t) \\ \dot{B}(t) = -(k_B + k_{AB})B(t) + k_{BA}A(t) \\ y(t) = \frac{A(t)}{V} \end{cases} \tag{1}$$

The nominal parameter $\theta^* = \begin{bmatrix} 0.5 & 0.25 & 1.1 & 0.75 \end{bmatrix}$. The model output $y(t)$ is simulated for $t = 0$ to $t = 3$ hours with the initial conditions $x_1(0) = x_2(0) = 0$ and the input $u(t)$ is unit impulse input given at $t = 0$.

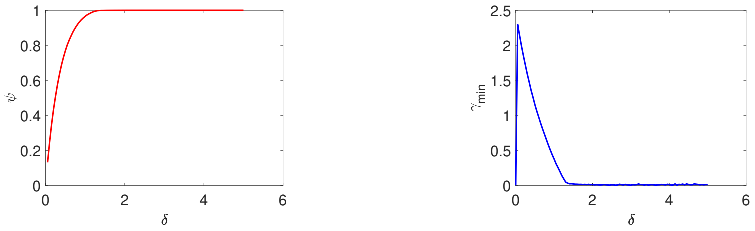

(a) δ vs γ plot (b) δ vs γ_{min} plot

Figure 2: (a) The curve hits unity value for $\delta > 1.6$ indicating local structural unidentifiability (b) The γ_{min} curve also indicate local structural unidentifiability because of zero value of $gamma_{min}$ for non-zero δ

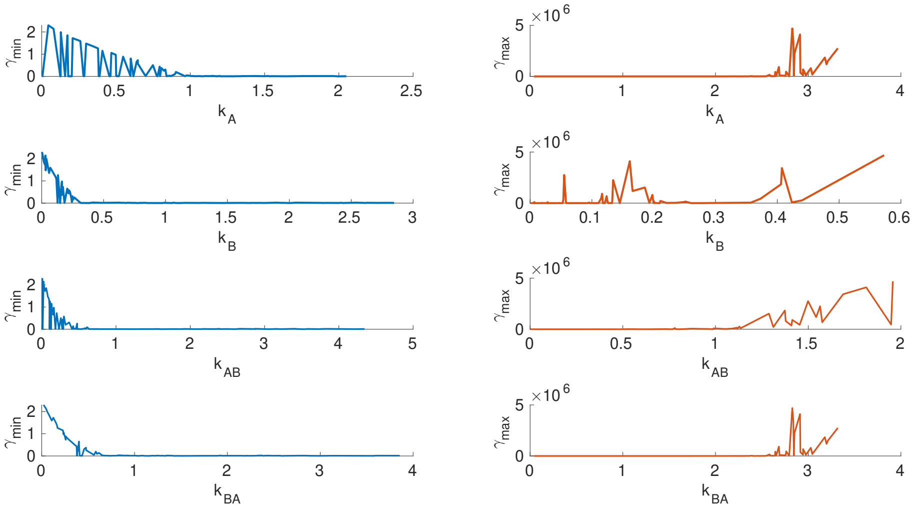

Figure 3: All the parameters have zero value γ_{min} and γ_{max} for non-zero δ_{theta_i}

From Figs. 2a and 2b, we can clearly see that the model sensitivity curve (ψ) hits unity value and $\gamma_m in$ hits zero value for non-zero delta indicating local structural identifibility. Further, to identify which parameters are unidentifiable δ_{θ_i} vs γ_{min} plot is generated in Fig 3. From Fig. 3 it is evident that all the parameters are unidentifbale for the given model and output pair. This result is consistent with the findings in [1].

2 High dimensional biochemical pathway model

A linear biochemical pathway with fourteen states, sixteen parameters and one input is considered for demonstration [3]. The states $x_1(t)$ and $x_{14}(t)$ are measured. The nominal parameter set (θ^*) is taken from [3].

$$
\mathcal{M}: \begin{cases}
\dot{x}_1 = -\frac{v_m x_1}{k_m + x_1} + p_1 u \\
\dot{x}_2 = -p_1 x_1 - p_2 x_2 \\
\dot{x}_3 = -p_2 x_2 - p_3 x_3 \\
\dot{x}_4 = -p_3 x_3 - p_4 x_4 \\
\dot{x}_5 = -p_4 x_4 - p_5 x_5 \\
\dot{x}_6 = -p_5 x_5 - p_6 x_6 \\
\dot{x}_7 = -p_6 x_6 - p_7 x_7 \\
\dot{x}_8 = -p_7 x_7 - p_8 x_8 \\
\dot{x}_9 = -p_8 x_8 - p_9 x_9 \\
\dot{x}_{10} = -p_9 x_9 - p_{10} x_{10} \\
\dot{x}_{11} = -p_{10} x_{10} - p_{11} x_{11} \\
\dot{x}_{12} = -p_{11} x_{11} - p_{12} x_{12} \\
\dot{x}_{13} = -p_{12} x_{12} - p_{13} x_{13} \\
\dot{x}_{14} = -p_{13} x_{13} - p_{14} x_{14} \\
y(t) = x_1(t) + x_{14}(t)
\end{cases} \quad (2)
$$

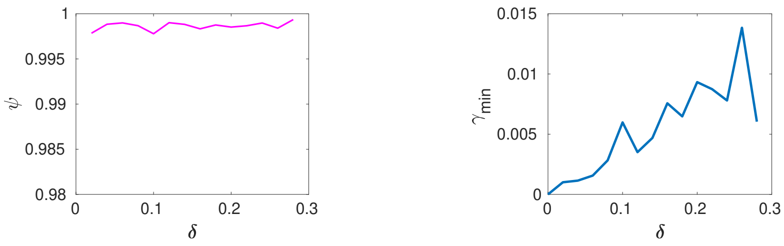

(a) δ vs ψ plot

(b) δ vs γ_{min} plot

Figure 4: (c) The curve does not hit the unity value indicating local structural identifiability. (b) The curve has significantly small slope and numerically small γ_{min} which indicate the sloppiness for the given δ

From Fig. 4b, the model is locally structurally identifiable. In Fig. 4a, the ratio of values of $\frac{\gamma_{max}}{\gamma_{min}}$ increases as δ increases; the ratio is numerically significant indicating the model is sloppy in the traditional and multi-scale notion of sloppiness. In addition to that, in Fig. 4b, the for $\delta > 0.25$, the $\gamma_{min} \approx 0.015$. This observation implies that the system is (ϵ, δ) sloppy, and a sub-set of insensitive parameters will

contribute to the sloppiness.

From Figures 5 to 8, it is observed that the parameters v_m, k_m, p_7 and p_9 are insensitive for $\delta_i > 0.03$ compared to other parameters. The sensitivity in maximum deviation direction is significantly high for the parameters p_5, p_6, p_{13} and p_{14} in the vicinity of θ_i^*. In sum, the biochemical network considered is structurally locally identifiable, and only 4 out of 16 parameters are insensitive for a particular region for the given δ. Further, the model has a moderate multi-scale sloppiness ratio inside the specified δ, which indicates an acceptable isotropic sensitivity in the parameter directions.

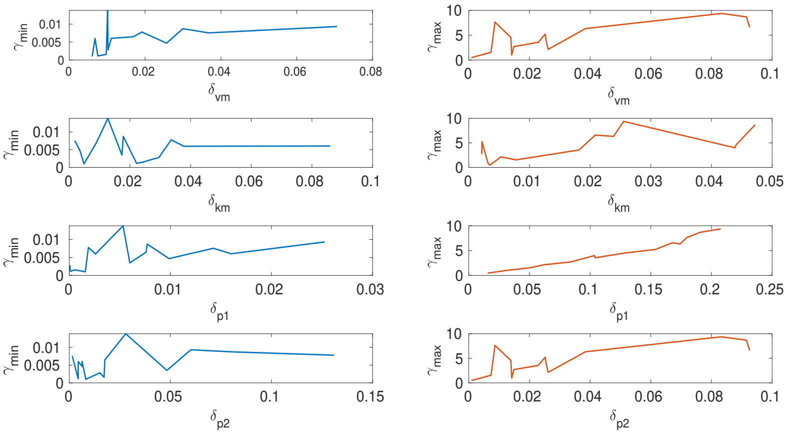

Figure 5: x-axis depicts the relative distance of the particular parameter θ_i from its reference value θ_i^*. y-axis on the left is the minimum sum-square deviation and on the it is the right maximum sum-square deviation from $y^*(t)$. Parameters v_m and k_m are insensitive for $\delta_i > 0.03$. The maximum sensitivity of parameter p_1 is linearly increasing as δ_i increases.

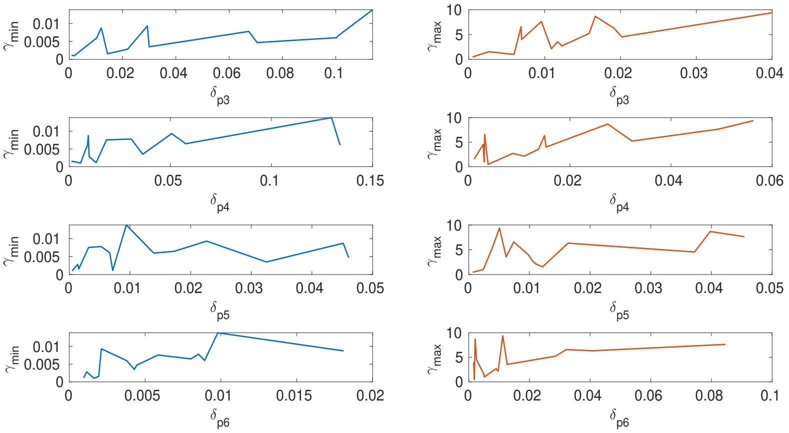

Figure 6: x–axis depicts the relative distance of the particular parameter θ_i from its reference value θ_i^*. y–axis on the left is the minimum sum-square deviation and on the right it is the maximum sum-square deviation from $y^*(t)$. While non of the parameters are insensitive, parameters p_5 and p_6 are highly sensitive in the vicinity of the $\theta_i^*(\delta_i^* < 0.01)$

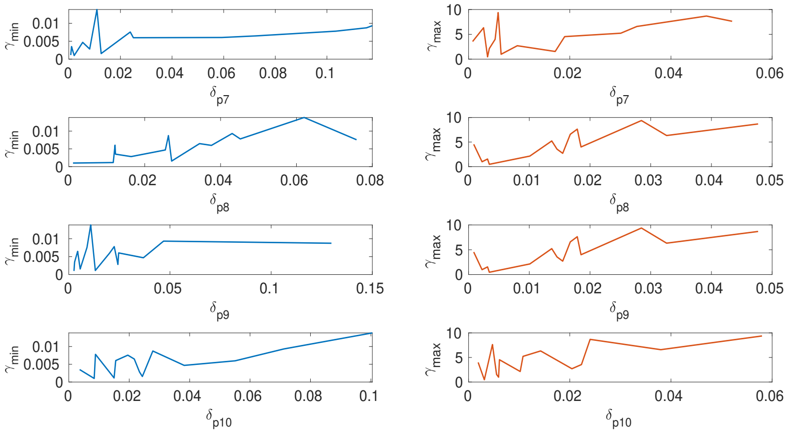

Figure 7: x–axis depicts the relative distance of the particular parameter θ_i from its reference value θ_i^*. y–axis on the left is the minimum sum-square deviation and on the right it is the maximum sum-square deviation from $y^*(t)$. The parameters p_7 is insensitive for $\delta_i > 0.02$ and p_9 for $\delta_i > 0.05$

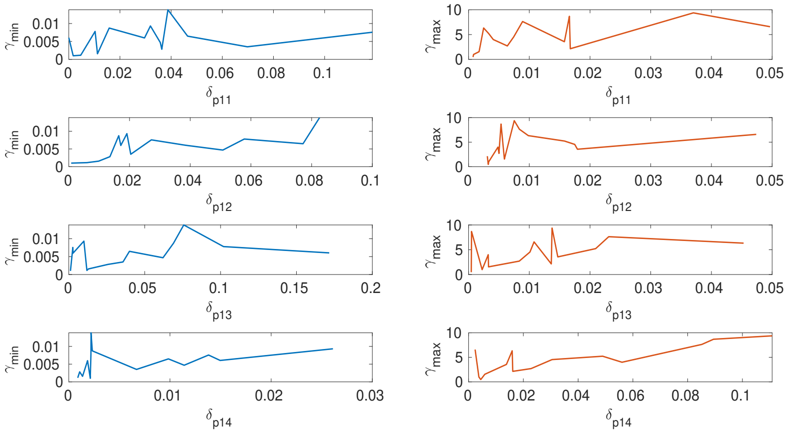

Figure 8: x-axis depicts the relative distance of the particular parameter θ_i from its reference value θ_i^*. y-axis on the left is the minimum sum-square deviation and on the it is the right maximum sum-square deviation from $y^*(t)$. The parameters p_{13} and p_{14} are highly sensitive in the vicinity of θ_i^*.

.

References

[1] Joseph distefano iii, dynamic systems biology modeling and simulation. *Science Progress*, 102(4):378–378, 2019.

[2] P J Nestel, H M Whyte, and D S Goodman. Distribution and turnover of cholesterol in humans. *J Clin Invest*, 48(6):982–991, June 1969.

[3] Oana-teodora Chis, Alejandro F Villaverde, Julio R Banga, and Eva Balsa-canto. Mathematical Biosciences On the relationship between sloppiness and identifiability. *Mathematical Biosciences*, 282:147–161, 2016.
