# Supplementary material for: Sloppiness: Fundamental study, new formalism and its application in model assessment
Source: PLoS One. 2023 Mar 8;18(3):e0282609. doi: 10.1371/journal.pone.0282609 (PMC9994762; doi:10.1371/journal.pone.0282609)
Supplement: S2 File — The ordinary differential equation models used in the examples and the link for the code for simulating the visual plot are provided in this supporting information file. (PDF) [file pone.0282609.s002.pdf]

Supporting information: Sloppiness: fundamental study, new formalism and quantification

Prem Jagadeesan[1,3,4], Karthik Raman[2,3,4*], Arun K Tangirala[1,3,4*]

[1]Department of Chemical Engineering, Indian Institute of Technology (IIT) Madras, Chennai – 600 036, India
[2]Department of Biotechnology, Bhupat and Jyoti Mehta School of Biosciences, IIT Madras, Chennai – 600 036, India
[3]Robert Bosch Centre for Data Science and Artificial Intelligence (RBCDSAI), IIT Madras, Chennai – 600 036, India
[4]Centre for Integrative Biology and Systems mEdicine (IBSE), IIT Madras, Chennai – 600 036, India

*kraman@iitm.ac.in, arunkt@iitm.ac.in

This section presents, the necessary model equations used for demonstration of the proposed method.

1 Pharmacodynamic HIV infection mode

The system is defined by four state variables and eight parameters as given below, where V stands for free virus (Model Output).

$$\mathcal{M}: \begin{cases} \dot{T_1} = s - \mu_T T + rT(1 - \frac{(T+T^*+T^{**})}{T_{max}}) - k_1 VT \\ \dot{T^*} = k_1 VT - \mu_T T^* - k_2 T^* \\ \dot{T^{**}} = k_2 T^* - \mu_b T^{**} \\ \dot{V} = N_v \mu_b T^{**} - k_1 VT - \mu_v T \end{cases} \tag{1}$$

2 Mitotic Oscillator

The dynamics of the Mitotic oscillator is governed by the following system differential equations. The system has three states and ten parameters.In the below equations, C denotes the cyclin concentration, while M and X represent the fraction of active cdc2 kinase and the fraction of active cyclin protease.

$$\mathcal{M}: \begin{cases} \frac{dC}{dt} = v_i - k_d C - v_d X \frac{C}{K_d + C} \\ \frac{dM}{dt} = \frac{V1(1-M)}{(K1+(1-M))} - \frac{V_2 M}{K_2 + M} \\ \frac{dX}{dt} = \frac{V3(1-X)}{(K3+(1-X))} - \frac{V_4 X}{X + K_4} \\ y(t) = C(t) + M(t) + X(t) \\ X(0) = M(0) = C(0) = 0.01 \end{cases} \tag{2}$$

3 Codes

The MATLAB code for constructing visual tool is provided in the following link `https://github.com/PremJ1704/My_Research/`
