# Supplementary material for: Sloppiness: Fundamental study, new formalism and its application in model assessment
Source: PLoS One. 2023 Mar 8;18(3):e0282609. doi: 10.1371/journal.pone.0282609 (PMC9994762; doi:10.1371/journal.pone.0282609)
Supplement: Attachment — Submitted filename: Response to the reviewers.pdf [file pone.0282609.s003.pdf]

Response to the Reviewers

Prem Jagadeesan, Karthik Raman and Arun K. Tangirala

December 29, 2022

The authors thank the reviewers for their valuable critical comments on the manuscript "Sloppiness: fundamental study, new formalism and its application in model assessment (Manuscript ID:PONE-D-22-27480R1)". The authors have taken into serious consideration of the reviewers comments and addressed them to the best possible extent. The authors believe that the reviewer's comments and the recommendations have greatly aided in improving the manuscript. The item by item response to the reviewer's comments is provided in the pages to follow.

Reviewer 1

1. *"rightly positioning the concept of sloppiness in relationship with identifiability."*
This is indeed an important point, but the manuscript does not adequately address the previous literature on the subject. In addition, to the cited papers, the authors should consider the relation of their work to following (and papers cited therein):

   Brouwer, Andrew F., and Marisa C. Eisenberg. "The underlying connections between identifiability, active subspaces, and parameter space dimension reduction." arXiv preprint arXiv:1802.05641 (2018).

   Evangelou, Nikolaos, et al. "On the parameter combinations that matter and on those that do not: data-driven studies of parameter (non) identifiability." PNAS Nexus 1.4 (2022):

   Response: We thank the reviewer for acknowledging an important point raised in the manuscript. We also thank the reviewer for pointing out the two references. The authors are aware of Brouwer and Eisenberg's work but unfortunately missed out on citing it. The revised version of the manuscript includes the relevant references (**LN:24-26** and **LN:93-97**)

   - Brouwer and Eisenberg (2018), have used average sensitivity FIM to perform global analysis on parameter sensitivity, sloppiness and identifiability. Further, they used the magnitude of eigenvalues and eigenvectors of sFIM to detect the sensitivity.
   - Given a nonlinear ODE, Evangelou *et al* (2022) developed a method to identify important and unimportant parameter combinations using manifold learning techniques (Dmaps). Further, they use a special type of autoencoder known as a Y-shaped conformal autoencoder to disentangle the unimportant parameter combinations. Finally, the identified effective parameters are mapped back to physical parameters.

2. *"elucidating the challenges in the current measure of sloppiness" By this statement, I understand the authors mean to clarify that sloppiness depends on the experimental conditions and observation structure. I argue that this fact was already implicitly stated in previous measure of sloppiness. Consider, for example, the Fisher Information measures. The FIM is calculated for specific measurements at specific experimental conditions, so measures of sloppiness must also depend on these things. However, the authors are correct that this has not been widely appreciated by the community and the fact bears clarifying and repeating.*

   Response: We thank the reviewer for appreciating the need for clarity on the prevalent understanding of the role of the model structure and data in sloppiness.

3. *"a new mathematical definition of sloppiness" In my opinion, this as the most significant contribution of the paper. The definition appears to be potentially useful and applicable to model analysis as demonstrated by the authors. However, I see some shortcomings in the proposed definition that are similar to problems with other proposed sloppiness measures. Previous measures have primarily used the condition number of the FIM or the approximate model Hessian as a measure of sloppiness. However this measure only accounts for total aspect ratio (eigenvalue spread) but does not quantify whether the eigenvalues are roughly equally spaced in log. In a similar way, the proposed definition accounts for total aspect ratio but does not measure a hierarchy of aspect ratios. I would like the authors to discuss these types of limitations.*

   Response: Firstly, we thank the reviewer for raising this concern. We partially agree with the reviewer. The (δ, ϵ) measure guarantees the existence of at least one sloppy direction. However, the proposed definition is a super-set of the existing definition in the following two important aspects,

- The proposed definition does not reflect the total aspect ratio. Rather, it defines the region of the parameter space over which model predictions are less than an arbitrarily small value (ϵ). The region obtained need not always be approximated to an n-ellipsoid; it may be approximately spherical, in which case the current measure of sloppiness might miss out on capturing it.
- The crucial problem in the traditional measure of sloppiness is that the notion of small or large eigenvalues needs to be better defined, as pointed out by Brouwer and Eisenberg. The prime reason is that the parameter estimates are affected by the eigenvalue magnitude and not the ratio. For an infinitesimal perturbation, the minimum eigenvalue of the FIM/Hessian of the cost is a function of δ as shown in Theorem 1. Moreover, the boundaries of the viable space of parameters dictate the magnitude of δ; by this, the user for a particular modelling exercise can assess the effect of eigenvalues on the precision of the parameter estimates.

Unfortunately, this clarity was missing in the manuscript. We regret the error. These points are now inserted in the revised version (**LN:342-353**).

4. *"a unified framework to assess sloppiness, structural identifiability, and parameter sensitivity". This contribution is really the consequence of contributions 1 - 3 and, given my concerns stated above, is overstated in the manuscript. I would describe the contribution as "improved methods to assess..." rather than a unified framework since the definitions proposed here are still incomplete.*

   Response: The word unified here refers to the point that a single method detects all three aspects of model quantification: sloppiness, identifiability and sensitivity. However, the authors also agree that the word improved method is more appropriate than describing it as a unified method. This change has been incorporated into the manuscript.

Specific comments

5. *I think the Cost function Eq. (4) is incorrect. There should be off-diagonal terms coupling different x's.*

   Response: The authors regret the mistake. The cost function has now been corrected. The implication of the corrected function on the sloppiness of the linear model has been modified accordingly in the text. However, the inference that the Hessian of the cost function is a pure function of data still holds. The subsequent use of Eq (4) on deriving the relationship between sloppiness and standard errors on the parameters has not been impacted since we have considered the inversion of diagonal elements of the Hessian as an estimate of variance (**LN:146**).

6. *Eq. 6 is missing signs in either the state space equations or the solution.*
   Response: The authors regret the mistake. The negative sign is now added to the Eq. (6) (state-space model) (**LN:158**).

7. *In Eq. 7, I believe the authors are reporting the Fisher Information and not the Hessian of the Cost.*
   Response: Eq. (7) is indeed the Hessian of the cost function derived with reference to Eq. (3) in Gutenkunst et al. Also, we agree that the Hessian of the cost function is equivalent to the Fisher Information matrix when the cost function is least squares and the data falls from a Gaussian distribution. However, since we have not considered the observation error in this model, we believe that the more appropriate way to mention Eq. (7) is as "Hessian of the cost function" than Fisher information.

8. *Figures 3 -4 would be clearer on log-scales.*
   Response: We thank the reviewer for the suggestion. However, in Figure 3, plotting the parameter space

and initial condition on a log scale makes the figure extremely skewed. For Figure 7, since the standard error range is less than unity, the figure loses interpretability while plotting in the log scale.

9. *Is the ratio in the definition of S (Eq. 8) backwards. As stated, S is small for a sloppy model, but I expect it should be the other way around.*

   Response: We understand the ambiguity faced by the reviewer. In the sloppiness literature, both the condition number and the inverse of the condition number have been used as measures. We adopted the inverse of condition number from Christian Tönsing *et al* (2014). The prime reason for adopting the inverse of the condition number is the interpretability in terms of identifiability. A model is known to be unidentifiable if the Hessian of the cost/ Fisher Information Matrix (FIM) is rank deficient, which again will result in zero value, the inverse of the condition number. Hence, the smaller the value of the inverse of the condition number, the model is more sloppy and closer to loss of identifiability.

10. *The new mathematical definition would be improved it were accompanied by a theorem showing a model sloppy by this definition will lead to an ill-conditioned FIM (previous definitions).*

    Response: We thank the reviewer for this suggestion. A theorem has been added to the section "New mathematical definition", showing the ill-conditioning of FIM by virtue of the proposed definition (**LN:320-351**).

11. *The literature review is missing all of the Sethna group's work from the last decade. A few papers the authors should cite and discuss include (and references therein):*

    Machta, Benjamin B., et al. "Parameter space compression underlies emergent theories and predictive models." Science 342.6158 (2013): 604-607.

    Transtrum, Mark K., et al. "Perspective: Sloppiness and emergent theories in physics, biology, and beyond." The Journal of Chemical Physics 143.1 (2015): 07B201_1.

    Response: Authors are aware of both the articles, unfortunately we miss out on citing them. The articles mentioned above and other relevant articles on sloppiness from the Sethna group has been cited in the manuscript. (**LN:71-72 and 83-88**).

    - Ben Matcha *et al* (2013) suggest that the sloppiness is essentially an effect of the multi-scale nature of the underlying process. Further, they show that specific models are not sloppy at microscopic scales, and sloppiness emerges only when fit to collective behaviour.
    - Transtrum et al (2015), suggest that the existence of sloppiness (dependence of model parameters to only a few macroscopic parameter directions) is responsible for the emergence of comprehensible macroscopic theories from highly complex microscopic processes.

Summary:

On the whole, the paper makes a marginal contribution to the question of sloppiness/identifiability analysis in systems biology. The literature review is inadequate and I have some concerns about the mathematical correctness of several equations and the clarity of presentation.

Response: The mathematical correctness of the Hessian of the linear model has been addressed. The rest of the equations are correct. With regards to the strength of contribution, the authors believe that the contribution is significant considering the enhancements that the current work brings to the definition, overcoming of crucial challenges in the existing measures and its potential application to model assessment.

Reviewer 2

1. *This is a generally well-written paper on a more formal approach of sloppiness which refers to the situation when the model output is sensitive to changes in so-called stiff parameters but largely insensitive to changes in sloppy parameters. The paper takes a further step towards formally defining sloppiness, and clarifying its relation with (practical) identifiability. The paper is well-structured and contains several useful examples illustrating the results. The results on linear models are also nice. However, there are some issues that need to be clarified before possible publication.*

   Response: The authors thank the reviewer for the positive feedback, and for pointing out the paper's key contributions. The authors agree with the issues mentioned, which have been carefully addressed in the revised version of the manuscript.

2. *The parameter estimation of nonlinear dynamical models is a classical field of electrical and control engineering with clearly defined notions from well before systems biology applications. This fundamental literature is largely missing from the paper (and also from most of the papers dealing with sloppiness.) A recent survey is e.g., (Schoukens, Johan, and Lennart Ljung. "Nonlinear system identification: A user-oriented road map." IEEE Control Systems Magazine 39.6 (2019): 28-99.)*

   Response: We agree with the reviewer. Challenges in parameter estimation have been systematically studied in the domain of control engineering. The reference mentioned above has been added to the manuscript now (**LN:47-49**).

3. *What is the benefit of sloppiness analysis in improving parameter estimation over more 'traditional' methods such as (practical) identifiability and sensitivity analysis?*

   Response: Sloppiness analysis characterizes the model's behaviour in an entire region in the parameter space, while identifiability and sensitivity analysis only focuses on bare parameter directions. One of the benefits of performing sloppiness analysis is that it helps to fix the initial values of a non-linear optimization algorithm, which directly impacts the goodness of the parameter estimates. As demonstrated in the article *"Why are nonlinear fits to data so challenging"* by *Transtrum et al.,* when the optimization gets struck in a sloppy region, the parameter estimates thus obtained will be biased and sub-optimal. This utility is mentioned in lines 491 to 494 in the main text.

4. *Table 1 contains the weighted Hessian of Cfor assessing sloppiness. This seems to contradict Table 2 where it is indicated that C does not influence sloppiness.*

   Response: We thank the reviewer for pointing out an interesting observation. The authors seek to draw the reviewer's attention to lines 201 to 205, where we have explained why the Hessian of the cost function has been traditionally used to assess sloppiness. A few portions have also now been rewritten for better clarity. Sloppiness, at its core, quantifies the sensitivity of the output with respect to the parameters and parameter directions. The influence of the cost function is indirect in that the change in the cost function will result in a different optimal estimate. However, it will not change the geometry of the gradient of the output with respect to the parameters/parameter directions. In Table 1, the cost function is used to assess sloppiness because the Hessian of the cost function is representative of the gradient of the output with respect to the parameters. The Hessian may not represent the gradient of the output when the cost function is changed.

5. *I cannot fully understand Eq. (9): there is θ_1 inside the norm but the $\forall\theta$ is used. Moreover, what is $\mathcal{S}$?*

   Response: We regret the confusion in the notation. θ_1 has been changed to θ. $\mathcal{S}$ is a set of all parameter vectors belong to the sloppy region. We regret missing the definition of $\mathcal{S}$. It has been added now (**LN:261-269**).

6. *In Definitions 1 and 2, it is not clear whether ϵ and δ are given when we use the defined notions. If so, why is it written that ϵ is arbitrarily small? Or do the definitions mean that for any ϵ, there exists δ such that the outputs will be closer than ϵ?*

   Response: We would like to clarify the meaning of the definition as follows.

   For a given model structure $\mathcal{M}$, the experimental condition $\mathcal{Z}$, for an arbitrarily small ϵ, if there exists a large δ, then, the model is sloppy. The magnitude of ϵ and δ are user-defined and vary with the needs of the modelling exercise. For example, ϵ may be chosen as the largest acceptable prediction error, and δ can be chosen as a function of an acceptable parameter range.

7. *Please comment on why the 2-norm is used in Definitions 1 and 2. (Other norms such as the infinity norm are also frequently used for parameter estimation.)*

   Response: We thank the reviewer for pointing out this insight. We agree that this definition holds for any distance measure. The reason for choosing *2-norm* is that it induces a nice spherical geometry in the parameter space, which is intuitive while assessing the model in the region of interest in the parameter space. Moreover, while sampling high-dimensional parameter space for model assessment, approximating the region of interest as an n-ball/n-sphere makes the sampling easier.

8. *I think the reference under Definition 2 should be Eqs. (9) and (10) instead of (7) and (9).*

   Response: We regret the mistake in referring to equation numbers. It has been corrected (**LN:270**).

9. *Why is Eq. (22) - which is an autonomous linear time invariant model - considered a non-linear predictor in the title of the section?*

   Response: The solution to the LTI model is considered the model's predictor equation and is nonlinear in parameters. Hence, the solution to the autonomous LTI model is regarded as a nonlinear predictor.
