# Supplementary material for: Sloppiness: Fundamental study, new formalism and its application in model assessment
Source: PLoS One. 2023 Mar 8;18(3):e0282609. doi: 10.1371/journal.pone.0282609 (PMC9994762; doi:10.1371/journal.pone.0282609)
Supplement: Attachment — Submitted filename: Response_to_reviewers.pdf [file pone.0282609.s004.pdf]

Response to Reviewers

Prem Jagadeesan, Karthik Raman and Arun K. Tangirala

January 31, 2023

Dear Prof. Csikász-Nagy,

Thank you for giving us the opportunity to further improve our manuscript "Sloppiness: fundamental study, new formalism and its application in model assessment" (Manuscript ID:PONE-D-22-27480R1) based on the reports that you received. We thank the reviewers for appreciating our contribution, and for the detailed reviews and constructive criticism. We have thoroughly revised the manuscript, based on the comments and suggestions put forth, addressing all points raised by the reviewers.

We have further included a point-by-point response to all the comments raised by both reviewers. In order to highlight the responses, we have included our reply to each specific suggestion the reviewer made, and how we addressed it in the revised manuscript.

We would like to thank the reviewers again for their thoughtful reviews, as well as the editor for handling this manuscript.

Sincerely, Prem Jagadeesan, Karthik Raman and Arun Tangirala

Reviewer 1

1. *I thank the authors for carefully considering and incorporating my suggestions. With the corrections to the equations and improved literature review, I believe the paper is technically correct. It contributes an interesting perspective to the sloppiness discussion. I recommend the paper for publication.*

   Response: We thank the reviewer for appreciating our contribution.

Reviewer 2

1. *The manuscript has been significantly improved since the initial version, and the most important notions are more clear in the revised paper. The authors have addressed all the critical issues raised in the review. The paper gives a useful step towards understanding the relations between identifiability and sloppiness notions. The only significant problem is that the claim and the proof of Theorem 1 are not precise enough.*

   Response: The authors thank the reviewer for acknowledging the changes in the revised version of the manuscript. The authors have addressed the reviewer's comments below.

   Major comments:

   (a) *What do the authors mean by "unique eigenvalues"? (Maybe distinct?).*

   Response: Yes, the reviewer's understanding is correct. The authors mean distinct eigenvalues. We regret for the confusion caused. The word "unique" has been changed to "distinct" now. (**Lines:329 & 341**)

   (b) *is* $\lambda_{min}(\nabla(C(\theta)))$ *actually* $\lambda_{min}(\nabla^2(C(\theta)))$*?*

   Response: Yes, the reviewer's understanding is correct. The authors regret the mistake. It has been corrected in the revised version. (**Line: 329**)

   (c) *Please explain* α.

   Response: α is an arbitrarily large scalar representing the length of the largest semi-axis of the hyper ellipsoid around the parameter on which the Hessian of the cost function is evaluated. α cannot be infinity because that results in a singular Hessian, which leads to the loss of identifiability. This explanation is now added into the manuscript (**Lines: 336-338**)

   (d) *Eq. (23) does not seem to be correct: The Hessian should be an* $n \times n$ *matrix. How can it be equal to a vector (difference of outputs)? Moreover, what is the index variable in the sums? Please clarify these issues.*

   Response: We regret the mistake in Eq. (23) and the subsequent derived equation Eq. (24). The equations are corrected in the revised version. The index variable sums over the observations. (**Lines: 331-332**)

   Theorem 1 and Corollary 1 aim to show that any model that is sloppy by Definition 1/Definition 2 will lead to an ill-conditioned FIM. For this, we show that at an infinitesimal perturbation from the optimal/reference parameter, the ϵ goes to zero, and the magnitude of minimum eigenvalue is δ. Further, δ approaches some arbitrarily large value α; then the minimum goes to zero, which in turn increases the sloppiness. Definition 1 and Definition 2 guarantee atleast one such sloppy direction.

   Minor Comments

2. *Example 1: From a slightly different point of view, it has been known that "it is possible for the linear predictor to show sloppiness for some experimental conditions". The condition to avoid this is called 'persistent excitation' in systems theory. See, e.g.: (Moore, J. B. (1978). On strong consistency of least squares identification algorithms. Automatica, 14(5), 505–509. doi:10.1016/0005-1098(78)90010) (Green, M., & Moore, J. B. (1986). Persistence of excitation in linear systems. Systems & Control Letters, 7(5), 351–360. doi:10.1016/0167-6911(86)90052) Moreover, such sloppiness can be an important sign of over-parametrization of the linear model compared to the information content of the measurements.*

   Response: Firstly, we thank the reviewer for the interesting insight. And we agree with the reviewer that with persistent excitation, sloppiness can be avoided in linear least-squares problems. However, from Eq. 4 and Eq. 5, it is seen that for any linear predictor, the source of sloppiness is data, and over-parametrisation can be seen as a consequence of fitting a higher order model when the data generating process is lower order. We also thank the reviewer for referring to the above interesting articles.

3. *Initial conditions may inlfuence even the structural identifiability of dynamical models. See, e.g.: (Saccomani, M. P., Audoly, S., & D'Angiò, L. (2003). Parameter identifiability of nonlinear systems: the role of initial conditions. Automatica, 39(4), 619-632.)*

   Response: We agree with the reviewer; the initial conditions and input may influence structural identifiability. The authors also cited the paper by Villaverde et al. on the role of input in structural identifiability ("Villaverde AF, Evans ND, Chappell MJ, Banga JR. Input-Dependent Structural Identifiability of Nonlinear Systems. IEEE Control Systems Letters. 2019;3(2):272–277."). For autonomous systems, the initial conditions can be seen as an impulse input at $t = 0$. However, while evaluating sloppiness, in this example, we have considered the subset of parameter space and experiment space (I.C.) over which the model is structurally identifiable. This is now mentioned in the revised manuscript (**Lines 166-167**), and the reference mentioned above is now cited in the manuscript **(Lines: 16-18)**.
